# The utility of diffusion‐weighted whole‐body imaging with background body signal suppression in detecting metastatic lesion of germ cell carcinoma

**DOI:** 10.1002/iju5.12327

**Published:** 2021-06-16

**Authors:** Kasumi Kaneko Yoshitomi, Noboru Numao, Yosuke Umino, Motohiro Fujiwara, Ryo Fujiwara, Tomohiko Oguchi, Yoshinobu Komai, Takeshi Yuasa, Shinya Yamamoto, Junji Yonese

**Affiliations:** ^1^ Department of Urology Cancer Institute Hospital of Japanese Foundation for Cancer Research Tokyo Japan; ^2^ Department of Urology Showa General Hospital Tokyo Japan

**Keywords:** diffusion magnetic resonance imaging, germ cell tumor, metastasis, radiotherapy, whole‐body imaging

## Abstract

**Introduction:**

Although the utility of diffusion‐weighted whole‐body imaging with background body signal suppression for assessing lymph node involvement or distant metastasis is renowned in many cancers, only few studies have revealed its utility for germ cell carcinoma. Some metastatic lesions of germ cell carcinomas are difficult to detect by conventional imaging.

**Case presentation:**

We report a case of a 70‐year‐old man with relapsed retroperitoneal germ cell tumor. Although his human chorionic gonadotropin levels increased, conventional imaging analysis showed no evidence of recurrence. Diffusion‐weighted whole‐body imaging with background body signal suppression was performed to search the metastatic lesion and detected metastatic sacral lesions. The patient responded well to local radiotherapy added to the steroid pulse and salvage chemotherapy and achieved long‐term recurrence‐free survival.

**Conclusion:**

Diffusion‐weighted whole‐body imaging with background body signal suppression has the potential to detect metastatic lesions not usually detected by conventional imaging methods.

**Abbreviations & Acronyms**
β‐HCG: beta human chorionic gonadotropin
ADC: apparent diffusion coefficient
CT: computed tomography
DWI: diffusion‐weighted imaging
DWIBS: diffusion‐weighted whole‐body imaging with background body signal suppression
FDG‐PET: fluorodeoxyglucose‐positron emission tomography
GCT: germ cell carcinoma
HCG: human chorionic gonadotropin
MRI: magnetic resonance imaging
T1WI: T1‐weighted imaging
T2WI: T2‐weighted imaging

**Keynote message**

DWIBS provides functional information on the entire body, and its utility for assessing lymph node involvement or distant metastasis has been widely recognized. Some metastatic lesions of GCTs are difficult to detect by conventional imaging (CT, bone scintigraphy, and FDG‐PET). Our findings suggest that DWIBS has the potential to detect metastatic lesions of GCTs that are not detected by conventional imaging.

## Introduction

DWIBS is a newly developed imaging modality, which is based on DWI, and provides functional information of the entire body.^1^ DWI visualizes the translational motion of water molecules, reflecting the cellular density of biological tissues and distinguishing malignancies from inflammatory changes.

The role of DWIBS in assessing lymph node involvement or distant metastasis has been widely recognized in many studies.^2^, ^3^, ^4^ However, for GCTs, only 1 study has investigated the utility of DWIBS. Mosavi *et al*. reported that DWIBS detected a normal‐sized lymph node metastasis of GCT showing high‐signal intensity; thus, it may be used as a feasible follow‐up imaging tool for GCTs.^2^

Herein, we report a case of relapsed GCT in which DWIBS could detect metastatic sacral lesions that were missed by conventional imaging, which contributed to effective local therapy.

## Case presentation

**Fig. 1:**
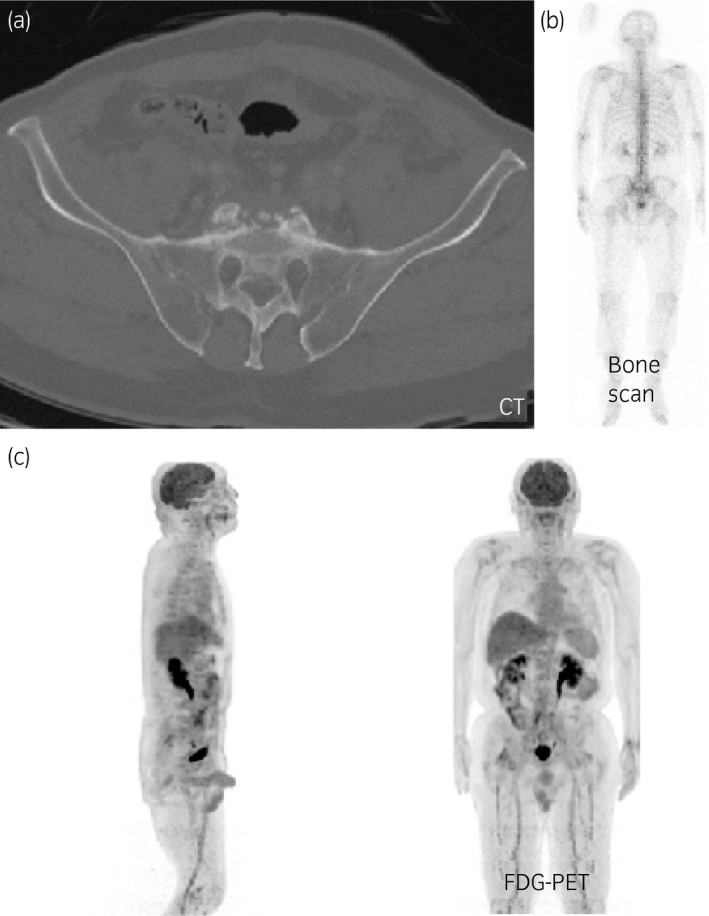
(a) CT, (b) bone scintigraphy, and (c) FDG‐PET findings when the re‐elevation of HCG levels was observed.

**Fig. 2:**
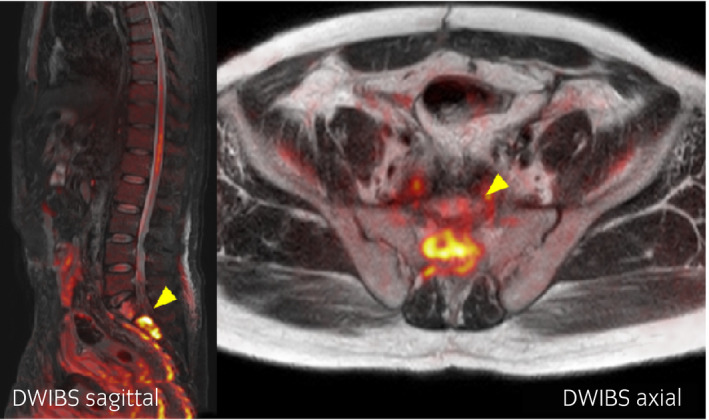
DWIBS findings when the re‐elevation of HCG levels was observed.

**Fig. 3:**
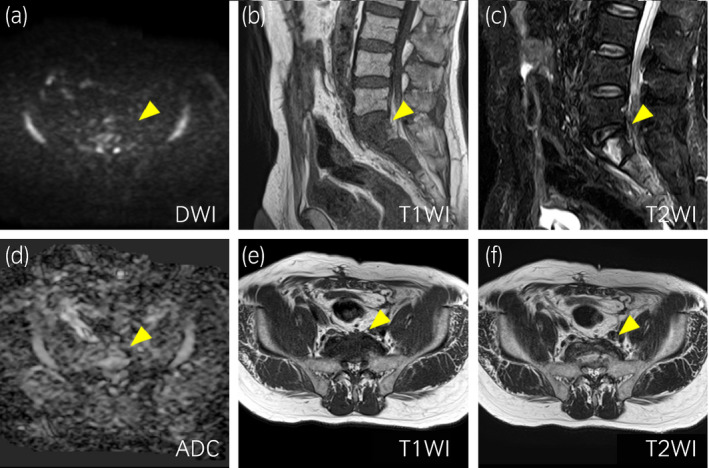
MRI findings when the re‐elevation of HCG levels was observed. (a) DWI, (b) sagittal T1WI, (c) sagittal T2WI, (d) ADC, (e) axial T1WI, (f) axial T2WI.

A 70‐year‐old man was referred to our hospital for further examination of an aortic bifurcation lymph node swelling of approximately 30 mm. *The bilateral testes were normal*. He had a history of non‐muscle invasive bladder cancer, but cystoscopy showed no evidence of recurrence. Clinical examination revealed no palpable lymphadenopathy. Cancer of unknown primary origin was suspected rather than metastatic bladder cancer. Tumor markers screening revealed high HCG (1990 mIU/mL) and β‐HCG (55 mIU/mL) levels. Other tumor markers, including alpha‐fetoprotein and lactate dehydrogenase, were within normal limits. As the swollen lymph node at the aortic bifurcation rapidly grew, open peritoneal lymph node biopsy was performed, and pathological examination revealed GCT with a histological pattern suggestive of a choriocarcinoma. Aortic bifurcation lymph node swelling further increased to 45 mm with bilateral common iliac artery and inferior vena cava invasion, multiple pulmonary metastases, and subclavian lymph node swelling. His HCG level increased to 7930 mIU/mL, and his β‐HCG level was 160 mIU/mL. He was clinically diagnosed with stage IIIB disease and was classified into the intermediate prognosis group according to the International Germ Cell Consensus Classification. After 4 courses of VIP chemotherapy (vincristine, ifosfamide, and cisplatin), 2 courses of TGP chemotherapy (cisplatin, gemcitabine, and paclitaxel), and 1 course of EP chemotherapy (etoposide and cisplatin), CT revealed 90% and 30% reductions of the metastatic and the primary lesion sizes, respectively. His HCG and β‐HCG levels were normalized. We dissected the residual mass at the aortic bifurcation with resection of the inferior vena cava and right common iliac vein. The pathological diagnosis confirmed choriocarcinoma with a small amount of residual viable cells. Adjuvant chemotherapy was not administered because of his old age and poor health status. After 4 months of chemotherapy, his HCG levels increased to 96.3 mIU/mL, but conventional imaging analysis (CT, bone scintigraphy, and FDG‐PET) showed no evidence of recurrence (Fig. 1). We then performed DWIBS, revealing an increased signal from the mass near the sacrum (Fig. 2). Spinal MRI revealed GCT recurrence with S1–S3 sacral invasion (Fig. 3). Then, the patient developed lower extremity pain, which was considered a neuropathic pain caused by GCT metastases. We started steroid pulse therapy immediately, and then administered radiotherapy (40 Gy/10 Fr) for the sacrum and salvage TGP chemotherapy. His tumor marker levels decreased to normal limits, and CT showed a remarkable reduction in the metastatic lesion size. Right lower extremity pain was reduced, and the patient achieved complete remission. Two years and 9 months after the last chemotherapy, CT showed that the metastatic lesion remained shrunk, and his tumor marker levels remained within normal limits. Informed consent was obtained from the patient for the publication of this case report and any accompanying images.

## Discussion

The role of DWIBS in assessing lymph node involvement or distant metastasis has been widely recognized. The greatest benefit of DWIBS is that it enables the evaluation of metastatic lesions in the whole body in 1 scan without any contrast medium. Enhanced CT, bone scintigraphy, and FDG‐PET required contrast medium administration. Furthermore, DWIBS can evaluate the systemic spread of malignant diseases without radiation exposure.^5^ In this case, relapsed retroperitoneal GCT with elevated tumor marker levels was not detected by conventional imaging. However, DWIBS detected a metastatic mass lesion near the sacrum. After the diagnosis of sacral metastasis on DWIBS, the patient developed lower extremity pain due to sacral metastasis. We started steroid pulse therapy and local treatment for sacral metastasis with radiation, preventing probable progression to lower limb paralysis.

DWIBS is superior to enhanced CT and FDG‐PET in localizing parenchymal neoplasms and identifying tumors with low glucose metabolism.^6^ Increased nuclear‐to‐cytoplasmic ratio and hypercellularity are generally observed in metastatic lesions, and DWIBS shows hyperintensity on metastatic lesions. Mosavi *et al*. suggested that signal intensity in DWIBS was used for evaluation of residual mass activity.^2^ Previous studies reported no correlation between glucose metabolism and water motion.^6^ We believe that DWIBS, which provides additional important information about metastatic lesions, is worth performing to detect metastatic GCTs.

An accurate detection of metastatic lesions in GCT patients is essential for an optimal therapeutic strategy; however, some metastatic lesions are difficult to detect by conventional imaging, including enhanced CT, bone scintigraphy, and FDG‐PET, as in this case. For example, patients with GCT limited to the testis on clinical staging but who have persistent tumor marker level elevation following orchiectomy are classified as having stage IS disease.^7^ However, persistently elevated tumor marker levels generally indicate the presence of metastatic lesions^7^; in fact, following radiological recurrence has often been observed.^8^ According to previous studies, 20–30% of the patients with stage IS disease had subclinical metastatic lesions mostly in the retroperitoneal lymph nodes.^9^, ^10^, ^11^ These findings suggest that metastatic lesion evaluation by conventional imaging can be associated with the risk of underdiagnosis.

There are some limitations to using DWIBS. DWIBS is inappropriate for evaluating residual masses post‐chemotherapy.^12^, ^13^ Chemotherapeutic drugs cause cell membrane rupture and cell necrosis, resulting in decreased cell density, thereby increasing water molecule mobility.^12^, ^13^ Therefore, tumors show high‐signal intensity and false‐negative results on DWIBS.^12^, ^13^ Second, the diagnostic accuracy of DWIBS in bone metastasis evaluation is controversial.^12^, ^14^ Metabolic changes affect water diffusion in the bone marrow and cause false‐positive results on DWIBS.^10^ Therefore, DWIBS should be integrated with conventional imaging analysis to assess metastatic lesions in the entire body.

## Conclusion

Our case suggests that, in GCT patients, DWIBS has the potential to detect metastatic lesions not detected by conventional imaging tools. Further studies should be conducted to reveal the diagnostic utility of DWIBS.

## Conflict of interest

The authors declare no conflict of interest.

## Ethics statement

Ethical approval of this study is not applicable.

## Approval of the research protocol by an institutional reviewer board

None declared.

## Informed consent

Written informed consent was obtained from all subjects for publication of this case report and accompanying images. A copy of the written consent is available for review upon requests.

## Registry and the registration no. of the study

None declared.
